# Research on Eye-Tracking Control Methods Based on an Improved YOLOv11 Model

**DOI:** 10.3390/s25196236

**Published:** 2025-10-08

**Authors:** Xiangyang Sun, Jiahua Wu, Wenjun Zhang, Xianwei Chen, Haixia Mei

**Affiliations:** 1 Key Laboratory of Intelligent Rehabilitation and Barrier-Free for the Disabled (Ministry of Education), Changchun University, Changchun 130022, China; sunxy@ccu.edu.cn (X.S.); 15645355660@163.com (J.W.); 2 School of Electronics and Information, Changchun University, Changchun 130022, China; 15153019931@163.com (W.Z.); silence8242025@163.com (X.C.)

**Keywords:** object detection, human–computer interaction, eye tracking control, eye tracking coding

## Abstract

Eye-tracking technology has gained traction in the field of medical rehabilitation due to its non-invasive and intuitive nature. However, current eye-tracking methods based on object detection technology suffer from insufficient accuracy in detecting the eye socket and iris, as well as inaccuracies in determining eye movement direction. To address this, this study improved the YOLOv11 model using the EFFM and ORC modules, resulting in a 1.7% and 9.9% increase in recognition accuracy for the eye socket and iris, respectively, and a 5.5% and 44% increase in recall rate, respectively. A method combining frame voting mechanisms with eye movement area discrimination was proposed for eye movement direction discrimination, achieving average accuracy rates of 95.3%, 92.8%, and 94.8% for iris fixation, left, and right directions, respectively. The discrimination results of multiple eye movement images were mapped to a binary value, and eye movement encoding was used to obtain control commands that align with the robotic arm. The average matching degree of eye movement encoding ranged from 93.4% to 96.8%. An experimental platform was established, and the average completion rates for three object-grabbing tasks controlled by eye movements were 98%, 78%, and 96%, respectively.

## 1. Introduction

Human–computer interaction (HCI) refers to the process of information exchange between humans and devices, with its core being the conversion between user intent and device response [1,2,3,4,5,6]. In recent years, HCI has developed rapidly, with new interaction methods emerging continuously. For example, voice interaction has been widely applied in smart devices. Zhu et al. [7] proposed a new scheme called SeVI, which adds an anti-eavesdropping mechanism to traditional voice interaction. Zhang et al. [8] proposed a strategy for HCI using physiological electrical signals—human triboelectricity (TEHB). By leveraging deep learning technology, the accuracy rate of text input directly obtained from handwriting can reach approximately 98.4%. Guo et al. [9] proposed using electromyography (sEMG) signals, with movement intent recognition serving as an intuitive input for human–machine interaction (HMI). Emerging brain-computer interface (BCI) [10,11] interaction technology can directly decode neural electrical signals generated by the human brain and convert them into control commands to operate external devices. Metzger et al. [12] created a neural prosthesis device for aphasia patients using an invasive BCI device, enabling users to spell at an average rate of 29.4 characters per minute. Eye-tracking interaction technology [13] has gradually moved toward practical application scenarios due to its advantages of being non-contact and non-invasive. Currently, commonly used eye-tracking signal acquisition and analysis devices primarily include eyewear-type and desktop-type eye trackers [14], which use near-infrared light technology to record the position of eye movements during motion [15]. For example, Wanluk et al. [16] utilized an eye tracker to obtain the direction of eye movement as input for an Arduino, enabling wheelchair movement control via eye direction. Niu et al. [17] proposed a click enhancement strategy to address issues such as low spatial accuracy in eye-controlled human–computer interaction. Wang et al. [18] proposed an eye-controlled human–computer interface (ECHCI) system, employing “gaze locking + blink triggering” as the interaction method in both temporal and spatial dimensions, and explored the optimization of blink-triggered actions. Niu et al. [19] proposed a reliable control triggering method, Magilock, effectively avoiding erroneous control triggers caused by multi-channel coordination mismatches and gaze point drift. Agarkhed et al. [20] designed a human–computer interaction system using eye-tracking technology, relying on detecting eye movements to understand user intent and performing corresponding operations based on the identified intent. Chen et al. [21] proposed a gaze-driven assembly assistance system that processes assembly activity video inputs using CNN and LSTM to identify assembly steps, while employing an eye tracker to estimate eye movement trajectories. In experiments, the system achieved an assembly step recognition accuracy of 98.36%, an average absolute error (MAE) of 4.37% for action counting, and an out-of-place recognition accuracy (OBOA) of 95.88%.

Object detection is a core task in the field of computer vision, aimed at accurately identifying, locating objects, and detecting their dimensions from images or video sequences [22]. Representative models include Fast R-CNN [23], Faster R-CNN [24], SSD [25], and YOLO, among others. As the application domains of object detection algorithms continue to expand, they have also found applications in areas such as autonomous driving and eye detection. For example, Wang et al. [26] proposed the YOLOv8-QSD network based on YOLOv8, a novel anchor-free driving scene detection network. This model achieved an accuracy of 64.5% on the large-scale small object detection dataset (SODA-A) while reducing computational requirements to 7.1 GFLOPs. Nguyen et al. [27] proposed a driver eye state monitoring system based on a lightweight convolutional neural network (CNN), achieving 25.11 FPS in real-time testing on a Jetson Nano device equipped with a 128-core NVIDIA Maxwell GPU. Liu et al. [28] explored the effectiveness of gesture interaction in driving assistance systems (DAS) through user research. Face detection is a critical component for efficiency in various fields such as security monitoring. Fredj et al. [29] developed a face detection implementation based on GPU components, optimizing memory access by leveraging different memory resources of the GPU. For detecting small targets, Xu et al. [30] proposed an improved small target detection model based on YOLOv5s, incorporating a multi-level feature fusion detection head, introducing a decoupled attention mechanism, and using a focus minimum distance intersection-over-union loss function. Yi et al. [31] developed an improved model, LAR-YOLOv8, based on YOLOv8. Experimental results on the NWPU VHR-10, RSOD, and CARPK datasets validated the model’s ability to detect small objects. Jain et al. [32] acquired eye images via a camera and combined them with an improved SSD algorithm to achieve iris detection of smaller objects. Eye detection is a fundamental task in important applications such as iris recognition in biometrics. Ruiz-Beltrán et al. [33] redesigned the Viola–Jones algorithm and proposed a hardware-based embedded real-time eye detection solution to address the high cost of current research systems. Nsaif et al. [34] proposed a hybrid enhanced eye detection method combining a region-based convolutional neural network (FRCNN) with Gabor filters (GNB) and a naive Bayes model. Experiments on the CASIA-IrisV4 database showed an average accuracy rate of 98% for eye detection. Chen et al. [35] proposed a coarse-to-fine, fast, and robust eye detector, effectively addressing the challenge of accurately detecting the eye and locating the pupil in captured images within video-based eye-tracking systems.

Addressing the issue of low accuracy in traditional eye-tracking control methods, previous researchers have proposed their own solutions, as mentioned in Reference [17]. In this study, we introduce an eye-tracking control method based on an improved YOLOv11 model, which enhances the model’s ability to capture eye movement information; a combination of frame voting mechanisms and eye movement area discrimination effectively improves the accuracy of eye movement direction discrimination; Eye movement encoding is used to generate control commands that are compatible with the robotic arm.

## 2. Method

### 2.1. Dataset Construction and Analysis

**Figure 1:**
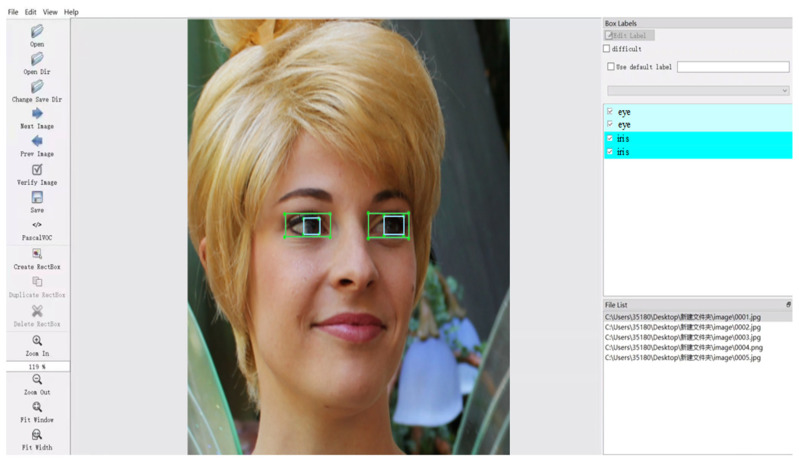
Images from the dataset annotated using the Labelimg tool in Python version 3.9.19.

This study requires simultaneous detection of the human eye socket and iris, but there are currently no publicly available annotated datasets. When constructing and annotating datasets for eye sockets and irises, factors such as ethnicity, age, whether glasses are worn, sample size, lighting and environmental conditions, and annotation rules must be considered for their impact on model performance. After comprehensive consideration, we selected the Human Faces dataset (Human-Faces Image Dataset) (https://www.kaggle.com/datasets/ashwingupta3012/human-faces, accessed on 7 May 2025) from Kaggle and annotated 8200 images using Labelimg, an open-source image annotation tool that supports the YOLO format, as shown in Figure 1. Rectangular boxes were added and category labels were specified to precisely annotate the images in the dataset. The rule is that the rectangular box must fully enclose the eye socket and iris. If there is partial occlusion, the unobstructed parts should be annotated and fully enclosed. Among these, 8000 images were used for training and testing the YOLOv11 model, with a training-to-testing ratio of 8:2.

**Figure 2:**
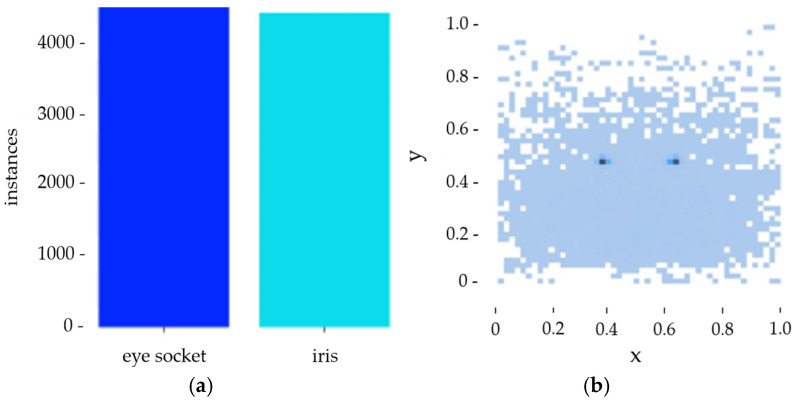
(**a**) Sample data bar chart. (**b**) Scatter plot of sample point location distribution.

Using MATLAB R2022a software to analyze the samples, the results shown in Figure 2a indicate that the sample distribution in this study’s dataset is relatively balanced. Figure 2b shows that the data points are distributed fairly evenly, so there is no problem of insufficient model learning due to uneven sample distribution and excessive differences in sample size.

### 2.2. Improving the YOLOv11 Model

#### 2.2.1. EFFM Module

**Figure 3:**
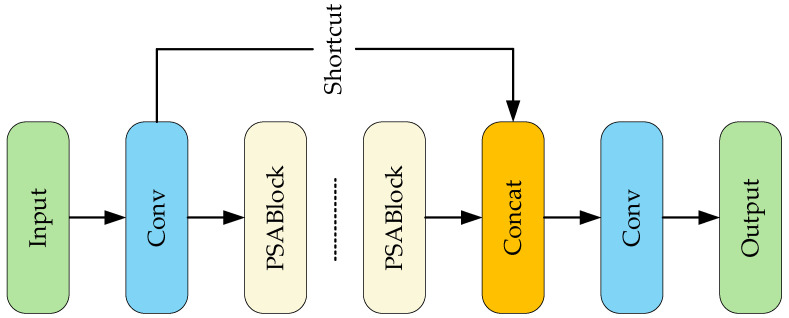
C2PSA module structure diagram.

The improvements of YOLOv11 over previous versions include the introduction of the C2PSA and C3K2 modules, with the C2PSA module structure shown in Figure 3. It uses Pyramid Squeeze Attention (PSA) to enable the model to focus precisely on the salient regions of an image. Multiple PSA blocks connected in series can enhance feature extraction. Shortcut connects the retained original information to achieve effective feature aggregation. Finally, feature fusion by the Concat module forms a high-quality feature representation.

However, in the original model, some feature maps were not enhanced by the C2PSA module. Directly concatenating them with other layers in the spatial dimension using Concat results in insufficiently refined features after fusion. To address this issue, this study designed an Enhanced Feature Fusion Module (EFFM) to replace Concat. This module integrates a branch-based multi-level attention mechanism with a complementary weighting mechanism to enhance feature fusion accuracy. The channel attention (Squeeze-and-Excitation Networks Attention, SE) [36] branch generates contextual information through global average pooling and dynamically assigns weights to each channel; The Spatial Attention (SA) [37] branch combines average pooling and max pooling feature maps to generate a spatial attention map highlighting significant regions in the enhanced image. Additionally, the weights obtained after processing through the attention module are not only directly applied to their corresponding feature maps but also interact with another input feature map to supplement each other’s information.

**Figure 4:**
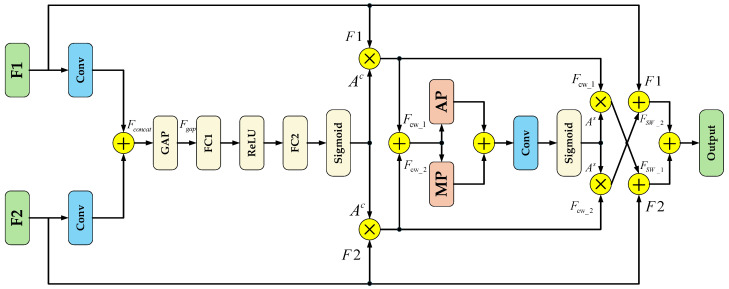
EFFM module structure diagram.

The EFFM module structure is shown in Figure 4. It takes feature maps F_1_ and F_2_ as input, extracts them through a convolution layer, and concatenates them in the channel dimension to form the preliminary fusion feature F_concat_, which provides the basis for fine-grained feature selection. Global average pooling (GAP) is applied to F_concat_ to generate a channel feature vector, capturing the overall channel information F_gap_. This helps strengthen the model’s perception of global semantics and provides global contextual information for the generation of channel attention; F_gap_ is processed through two fully connected layers using ReLU and Sigmoid activation functions to obtain the channel attention weights A^C^, enabling the model to adaptively adjust the contribution of each channel, as shown in Equation (1), where σ represents the Sigmoid function, FC_1_ is the first fully connected layer, and FC_2_ is the second fully connected layer.

(1) Ac=σFC2ReLUFC1Fgap

After applying A^C^ to the initial features, we obtain the channel-weighted features F_CW_1_ and F_CW_2_, as shown in Equation (2). ⊗ represents element multiplication. Channel weighting of the original feature map enhances the model’s selective expression capability in the channel dimension.

(2) $$F_{\mathrm{cw}\_1}=A^{c}⊗F_{1},\;F_{cw\_2}=\left(1-A^{c}\right)⊗F_{2}$$

Concatenate F_CW_1_ and F_CW_2_, then obtain spatial aggregation features by capturing significant regional information through maximum pooling (MP) and extracting global background information through average pooling (AP). Concatenate them again in the channel dimension to obtain F_spatial_concat_, as shown in Equation (3).

(3) $$F_{spatial\_caoncat}=Concat\left(MP\left(Concat\left(F_{cw\_1},F_{cw\_2}\right)\right),AP\left(Concat\left(F_{cw\_1},F_{cw\_2}\right)\right)\right)$$

After passing through the F_spatial_concat_ input convolutional layer, the spatial attention weights A^S^ are generated via the Sigmoid activation function, capturing the prominent regions of spatial information and enabling the model to adaptively emphasize important spatial regions in the feature maps; applying A^S^ to the channel-weighted features yields F_SW_1_ and F_SW_2_, as shown in Equation (4), which enhances the expressive power and discriminative ability of the features under the dual attention of channels and spatial regions; Finally, the features F_SW_1_ and F_SW_2_ are respectively complementarily weighted with the original inputs F_1_ and F_2_, and combined to generate the final output.

(4) $$F_{SW\_1}=F_{CW\_1}⊗A^{s},F_{SW\_2}=F_{CW\_2}⊗A^{s}$$

#### 2.2.2. ORC Module

**Figure 5:**
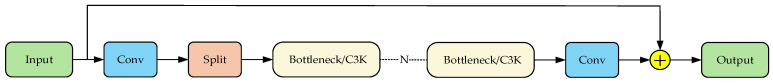
C3K2 module structure diagram.

The C3K2 module in YOLOv11 is primarily responsible for feature extraction, with its most important parameter being C3K, which can be set to False or True. These settings correspond to using the Bottleneck or the C3K module internally within C3K2, respectively, and the number of Bottleneck or C3K modules can be adjusted via the parameter N. As shown in Figure 5, C3K2 divides the input feature data into two branches for processing and then concatenates the output data. Each module has its own advantages. Compared to C3K, the Bottleneck module’s convolutional layers have fewer parameters, effectively reducing computational complexity and achieving a balance between coarse-grained and fine-grained feature representations; while the C3K module can use convolutional kernels of different sizes, enabling the model to extract features at different scales and helping to capture more complex and deeper spatial features.

**Figure 6:**
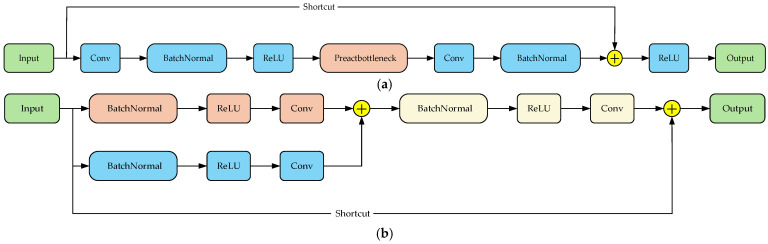
(**a**) ORC module structure diagram. (**b**) Preactbottleneck module structure diagram.

In practical applications, model lightweighting is of critical importance. The EFFM module increases the number of model parameters. While the C3K2 module of the original model can reduce some computational load and parameter count during the low-level feature extraction stage, it still suffers from issues such as feature redundancy and low computational efficiency. To address this, this study combines the ResNetv2 pre-activation design [38] with the RepVGG reparameterization concept [39] to design the ORC module as a replacement for C3K2. The module structure is shown in Figure 6a. The input data first undergoes feature extraction through a 3 × 3 convolution layer, followed by batch normalization and ReLU activation to complete the nonlinear activation of features. The pre-activation bottleneck (Preactbottleneck) module is then used for further feature extraction, with a 1 × 1 convolution layer for dimension adjustment. Finally, the processed features are added to the original features and undergo final activation processing via the ReLU activation function. The structure of the Preactbottleneck module is shown in Figure 6b, where the orange path represents the pre-activation path and the blue path represents the reparameterization path. The pre-activation path uses a 3 × 3 convolution kernel to extract spatial features, focusing more on basic feature extraction and gradient optimization before activation. The reparameterization path introduces a 1 × 1 convolution operation to achieve linear combination of features. After merging the two paths, a 1 × 1 convolution is used to extract the information contained within to achieve feature enhancement.

### 2.3. Eye-Tracking Control Method Based on an Improved Model

#### 2.3.1. Eye Movement Area Discrimination

**Figure 7:**
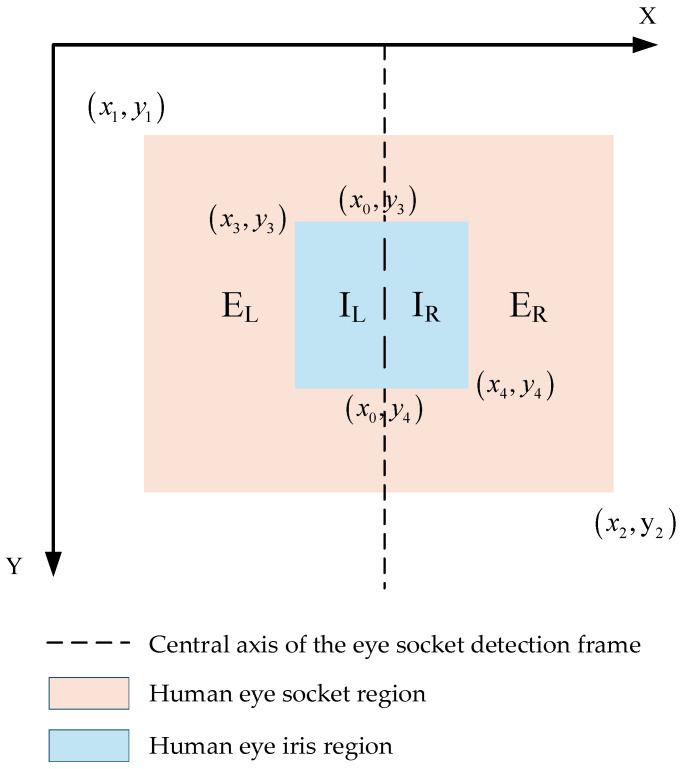
Eye movement area discrimination effect diagram.

This study uses the human eye socket and iris as the basic elements for determining eye movement direction. Since the iris has a relatively large range of movement in the horizontal direction, the results are categorized as leftward, rightward, or fixation in the horizontal direction. However, since part of the iris may be obscured by the eyelid, in such cases, the model can only detect partial iris regions and cannot accurately capture the actual coordinates of the center point of the iris detection frame. This renders the method of determining the left-right movement direction of the iris by calculating the distance from the center point to the left and right sides of the eye socket unreliable. Therefore, this study proposes a new method for eye movement direction determination based on eye movement area. This method divides the eye region using the central axis of the eye socket detection box and determines the eye movement direction by calculating the area of the iris in the two regions. The eye coordinate information detected by the improved model from a single frame image is shown in Figure 7, (x_1_,y_1_) is the upper-left coordinate of the orbital region, (x_2_,y_2_) is the lower-right coordinate of the orbital region, (x_3_,y_3_) is the upper-left coordinate of the iris, (x_4_,y_4_) is the lower-right coordinate of the iris, and the coordinates of the central axis of the orbital detection frame are (x_0_,y_3_) and (x_0_,y_4_). The central axis divides the orbital and iris detection frames into the left orbital region E_L_, the right orbital region E_R_, the left iris region I_L_, and the right iris region I_R_.

**Figure 8:**
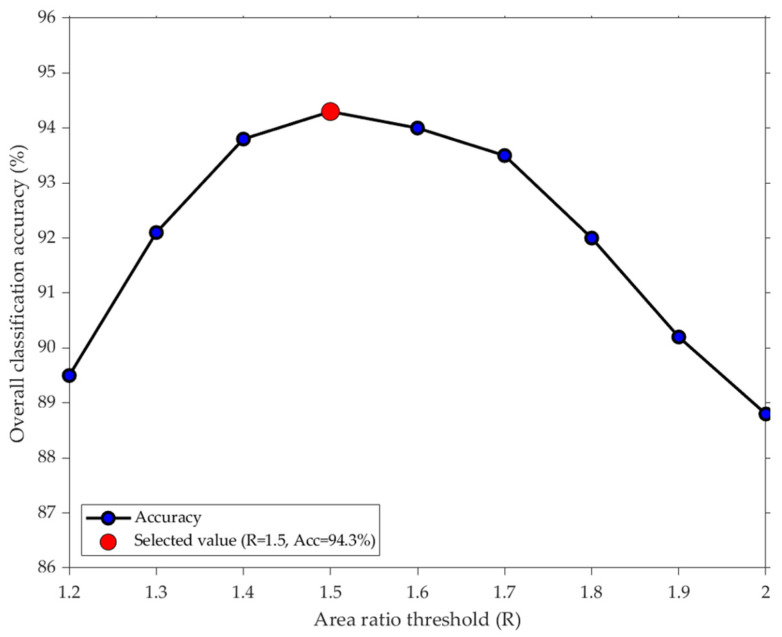
Results of the selective ratio threshold test experiment.

In subsequent experiments, control commands are encoded based on eye movement direction, making the accuracy of eye tracking critical. Strict constraints must be imposed on the relationship between S_IL_ (area of I_L_) and S_IR_ (area of I_R_). Considering that excessive parameters could compromise the real-time performance of subsequent eye-tracking control, a proportional relationship is adopted to limit the size ratio between S_IL_ and S_IR_. When the eyes gaze straight ahead, the iris is centered, and the left and right areas are approximately equal (ratio of about 1.0). During eye movements, this ratio deviates from 1.0. If the adopted ratio is too small (e.g.,1.2), the system becomes overly sensitive, prone to misinterpreting minor eye tremors as valid commands. Conversely, if the ratio is too large (e.g.,2.0), the system becomes sluggish, requiring substantial eye movements for recognition, leading to some valid eye movements being misclassified as fixations. Therefore, using the MATLAB software platform, this study conducted testing experiments on the annotated test dataset. The eye movement direction discrimination method was run at ratio values ranging from 1.2 to 2.0 in increments of 0.1, and the overall classification accuracy corresponding to each value was calculated. As shown in Figure 8, the accuracy curve forms a high and relatively stable plateau within the restricted ratio range of 1.4 to 1.7. The 1.5 threshold lies near the peak of this plateau, achieving approximately 94% accuracy. Thus, the ratio can be set to 1.5 at the data level. If iris displacement fails to meet the standard, it is classified as a gaze. Here, S_IL_ and S_IR_ can be calculated using Equations (5) and (6), while eye movement direction classification involves multiple scenarios, which are presented in Table 1.

(5) $$S_{IL}=\max\left(0,\;\left(x_{0}-x_{3}\right)\times \left(y_{4}-y_{3}\right)\right)\;$$

(6) $$S_{IR}=\max\left(0,\left(x_{4}-x_{0}\right)\times \left(y_{4}-y_{3}\right)\right)\;$$

The eye movement area discrimination method proposed in this study evaluates the area occupied by the iris in the left and right parts of the eye socket. It has stronger fault tolerance for situations where the detection frame cannot completely contain the iris. Even if part of the iris is not visible, it can still make a relatively stable eye movement direction judgment based on the distribution of the remaining part in the area. Since subsequent eye movement encoding is based on binary values, the eye movement direction discrimination results are represented by three labels: 0 for left, 1 for right, and None for fixation.

#### 2.3.2. Eye Movement Area Discrimination Method Combined with Frame Voting Mechanism

The eye movement area discrimination method is used to determine the direction of eye movement in a single-frame image. However, during real-time detection, when head displacement or object obstruction occurs, the detection frame may only include part of the iris and orbital area, leading to errors in the eye movement direction discrimination results. To address this issue, this study proposes an eye movement direction discrimination method that combines frame voting mechanisms with eye movement region area discrimination. This method consists of two parts: filtering invalid frames and statistical voting output. Filtering invalid frames involves screening the eye socket and iris coordinates and confidence information provided by each frame image. Frames with incomplete coordinates or confidence below 0.5 are deemed invalid and discarded, while frames with complete coordinate information and high confidence are deemed key frames and proceed to the next step. Statistical voting output processes the classification results of multiple key frame images within the N (voting time window). Specifically, it counts the number of labels N_0_ and N_1_ for different eye movement directions, maps the most frequent label to a binary value, where fixation (None) is not counted. If N_0_ > N_1_, the output is 0; if N_0_ < N_1_, the output is 1; if N_0_ = N_1_, the output is invalid, discard this set of data and proceed with the analysis of the next set of data.

**Figure 9:**
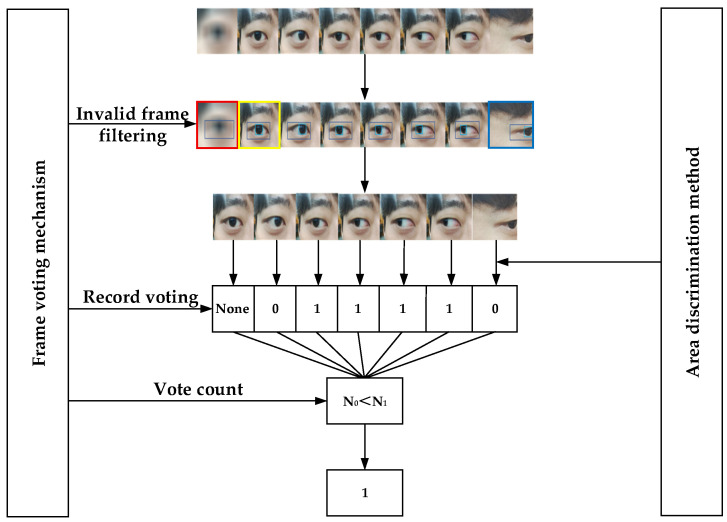
Flowchart of the eye movement area discrimination method combined with the frame voting mechanism.

Taking the example of obtaining 8 frames of images within each N, the method flow is shown in Figure 9. The detection model obtains information from each frame of images, filters out invalid frame information, and outputs eye movement direction labels (0, 1, None), etc. After these steps, the label counts N_0_ = 2 and N_1_ = 4 are obtained, and based on the predetermined conditions, the output is determined to be 1 (right direction). Among these, the image results highlighted in yellow in the figure are deemed as fixation, and the None label does not participate in the voting record; the images highlighted in red are deemed invalid frames and discarded due to camera focusing issues that prevented iris detection, with a confidence level below 0.5; the images highlighted in blue are misjudged as 0 (left direction) due to head displacement.

#### 2.3.3. Eye Movement Encoding and Command Design

**Figure 10:**
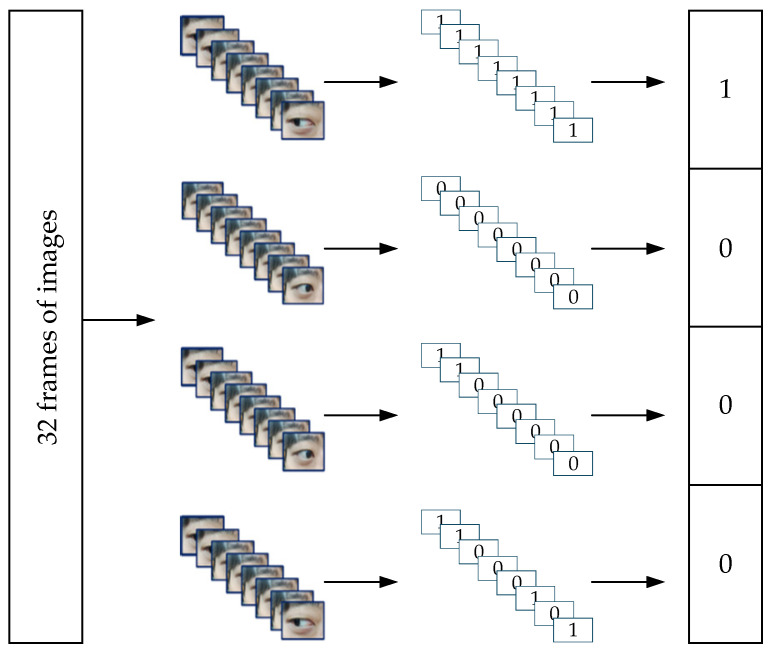
Eye movement encoding schematic diagram.

Due to the limited range of eye movements, there will be a shortage of commands in subsequent control. Eye movement encoding is an effective way to expand the number of commands. This method maps eye movement directions to basic control commands, and further combinations can yield more commands. Binary codes have the advantages of concise representation and low transmission overhead. Therefore, this study uses 4-bit binary codes as control commands to control the robotic arm. The principle of eye movement encoding is illustrated in Figure 10. Taking a video stream with 32 frames as an example, each time window yields 8 frames of images. After obtaining the iris and orbital coordinates of each frame and filtering out invalid frames, the eye movement direction is determined based on the information from key frames. Finally, the eye movement direction with the highest proportion across the 8 frames is selected through a voting process, and the corresponding label is output. This process completes the processing of the video stream, yielding the corresponding 4-bit binary code.

This study ultimately aims to apply eye-tracking control commands to a robotic arm system for experimentation. Therefore, we designed eye-tracking control command functions as shown in Table 2. Users can obtain a 4-bit binary code through iris movement and use it to control the robotic arm’s eight movements. Since we have implemented a mechanism that automatically ends the experiment if no target is detected within 20 s, there is no need to design an end command; the confirmation command is critical for preventing erroneous responses during eye-tracking control, and movement commands must be combined with it to take effect; the start command initiates the program and does not need to be combined with the confirmation command.

### 2.4. Set up an Experimental Platform

#### 2.4.1. Physical Implementation of Platform Movement

The experimental platform used in this study is the Elit EC66 robotic arm. The position of the robotic arm in space is described using a Cartesian coordinate system composed of three vertical coordinate axes. Considering that achieving precise positioning of the robotic arm through fixed positions alone cannot meet the requirements for control flexibility, we adopt an incremental movement approach. By setting the offset value σ, the robotic arm can perform incremental movements in different directions. The value of σ must be set in the robot’s programming language, JBI. The offset direction is controlled by the coils of the EC66 main controller, with the coil functions detailed in Table 3. Assigning the 4-bit binary eye-tracking control commands to coils M531–M534 enables precise control of the robot’s motion.

The coil function codes are shown in Table 4 and correspond one-to-one with the eye control commands in Section 2.3.3. Among them, the functions corresponding to 0101 and 1010 are independent of the offset direction and instead trigger the Socket program TOOLCLOSE and TOOLOPEN for the robotic gripper.

#### 2.4.2. Implementation of the Platform’s Data Transmission Protocol

**Figure 11:**
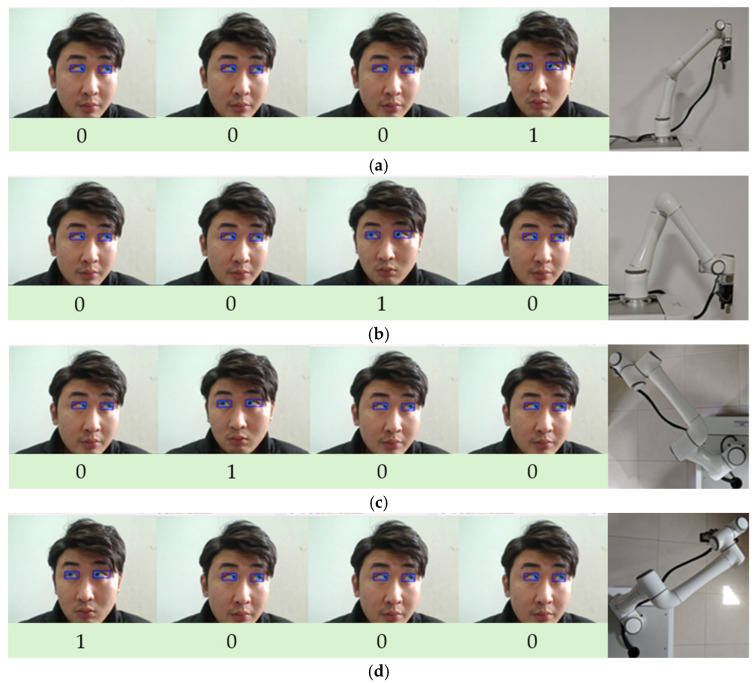
(**a**) Eye-controlled robotic arm moves upward; (**b**) The robotic arm moves downward; (**c**) The robotic arm moves to the left; (**d**) The robotic arm moves to the right.

Considering the convenience of the experimental platform, this study uses a local area network communication method to achieve data transmission between the PC and the robotic arm. The robotic arm’s static IP address is configured as “192.168.10.9,” and the port number is set to “7777.” The router was set to 10 frequency bands, and the PC’s IPv4 address range was modified to 10. The router was connected to the robot arm’s open communication port via an Ethernet cable, and communication used the TCP/IP protocol, with information transmitted in TCP/IP standard packet format. The platform must pre-set the time window parameters. For example, if the time window is set to 1 s, the user must complete the eye movement within 1 s after the command recognition begins. The system will save the output label. Through this process, the four-bit binary code is encoded into an eye movement control command and sent to the robotic arm. Figure 11 shows the implementation effect of controlling the robotic arm to move in four directions: up, down, left, and right. Each eye socket and iris detection image in the figure represents the eye movement direction label determined by analyzing multiple frames of images.

## 3. Experimental Results and Analysis

### 3.1. Human Eye Socket and Iris Recognition Experiment

#### 3.1.1. Experimental Environment and Parameter Settings

All experiments in this study were conducted using the PyTorch 2.0.1 deep learning framework. Before the experiments, the initial learning rate was set to 0.001, Mixup was set to 0.0, Paste_in was set to 0.0, Batchsize was set to 8, and the total number of iterations was set to 300 rounds. The specific experimental environment is shown in Table 5.

#### 3.1.2. Model Comparison Experiment

To validate the performance of the improved model in this study, we conducted comparative experiments with other mainstream models on the same dataset. Before the comparison, we selected precision, recall, average precision (AP), and mean average precision (mAP) as evaluation metrics for the human eye socket and iris recognition model; Among these, mAP uses mAP@0.5, which is the mean average precision at an Intersection over Union (IOU) threshold of 0.5. This serves as the evaluation standard for the model’s overall performance, with higher mAP@0.5 values indicating superior overall model performance. The experimental results are shown in Table 6.

The experimental results show that the improved model in this study performs excellently in terms of accuracy, recall rate, and mAP@0.5, with mAP@0.5 reaching 0.92, which is 46.5% higher than SSD and 39.8% higher than Faster R-CNN. This proves that the model has improved in terms of reducing false negatives and false positives of target objects.

#### 3.1.3. EFFM Module Comparison Experiment

The EFFM module is formed by introducing a complementary weighting strategy based on the attention mechanism. This module aims to enhance model performance. To validate the effectiveness of the EFFM module, we compared it with the attention mechanisms commonly used in YOLO models: SE [40], CA [41], and BiFormer [42]. The results are shown in Table 7. The YOLOv11+BiFormer model achieves the highest recall among all methods at 0.862. However, compared to the YOLOv11+EFFM model, its recognition accuracy and mAP@0.5 are 0.9% and 0.3% lower, respectively. Since mAP@0.5 is a key metric for evaluating overall model performance, the YOLOv11+EFFM model demonstrates superior comprehensive performance compared to YOLOv11+BiFormer. The model incorporating the EFFM module outperforms those with CA, SE, and BiFormer attention mechanisms by 16.5%, 15%, and 0.3% respectively in mAP@0.5. This clearly indicates that the use of the EFFM module significantly enhances model performance.

#### 3.1.4. Ablation Experiment

An ablation experiment is an experimental method that involves sequentially removing certain modules from a model to observe changes in its performance metrics. In this study, ablation experiments were conducted to validate the impact of the two modules we designed on the performance of the YOLOv11 model. Using YOLOv11 as the baseline model, Experiment 1 replaces the Concat module with the EFFM module, Experiment 2 replaces the C3K2 module with the ORC module, and Experiment 3 replaces the original modules with both the EFFM and ORC modules.

The experimental results are shown in Table 8. The average accuracy of the base model in identifying eye socket categories is 0.927, which is good, but the recall rate and average accuracy in identifying iris categories are 0.421 and 0.511, respectively, indicating that the model has serious problems in identifying iris category samples.

Experiment 1 shows that the use of the EFFM module improves the model’s accuracy in recognizing the eye socket and iris by 2.8% and 9.5%, respectively, compared to the baseline model. The recall rates are improved by 6.1% and 33.7%, respectively, and the mean average precision (mAP@0.5) across all categories is improved by 19.1%.

Experiment 2 shows that after using the ORC module, the model’s accuracy for eye socket and iris recognition improved by 2.4% and 4.6%, respectively, compared to the baseline model. The recall rate for eye socket categories decreased by 2.4%, while that for iris categories increased by 4.6%. The mAP@0.5 for all categories improved by 4%. Although the performance improvement is smaller compared to the EFFM module, the ORC module can enhance recognition accuracy while reducing computational costs. As shown in Table 9, after using the ORC module, the number of parameters decreased by 105,056 compared to the baseline model, and the computational cost (GFLOPs) decreased by 3.1%.

Experiment 3 shows that when the EFFM module and ORC module are used together, the model’s accuracy for orbital and iris recognition improves by 1.7% and 9.9%, respectively, compared to the baseline model, while recall improves by 5.5% and 44%, respectively. The mAP@0.5 for all categories improves by 20.1%. From the perspective of computational cost, compared to the baseline model, the number of parameters decreased by 73,952, and the computational cost decreased by 1.56%, second only to the model in Experiment 2.

#### 3.1.5. Comparison of Experiments Before and After Model Improvement

**Figure 12:**
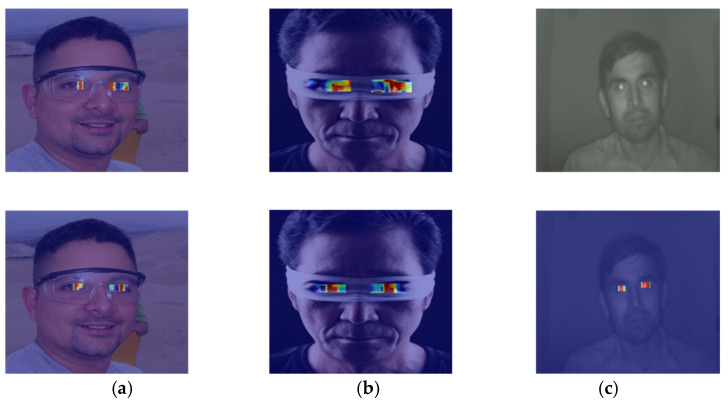
Heat map comparison experiment diagram. (**a**) shows the state when wearing glasses, (**b**) shows the state when part of the eye is covered, and (**c**) shows the state in low light conditions.

In object detection tasks, heatmaps reveal the areas of focus during model decision-making, enhancing the model’s interpretability. Grad-CAM (Gradient-weighted Class Activation Mapping) is a method that generates heatmaps based on gradient information from the last convolutional layer of a convolutional neural network, using color changes to indicate the model’s level of attention to these areas. The closer the color is to red, the higher the model’s attention to that area. This study employs this method to conduct heatmap comparison experiments on the model before and after improvement, using images under different conditions as input. The experimental results are shown in Figure 12, where the first row displays the heatmaps generated by the original model, and the second row shows the heatmaps generated by the improved model.

Group (a) represents the scenario where glasses are worn. In the improved model’s heatmap, the red areas are concentrated around the iris and provide more comprehensive coverage; Group (b) represents the scenario where the eye is partially obstructed. In the improved model’s heatmap, the red areas are closer to the actual location of the iris; Group (c) represents the scenario under low-light conditions. The original model does not focus on the eye area, while the improved model can effectively concentrate attention on the eye area, particularly the iris.

### 3.2. Eye Movement Discrimination and Coding Method Experiment

#### 3.2.1. Eye Movement Direction Discrimination Accuracy Experiment

**Figure 13:**
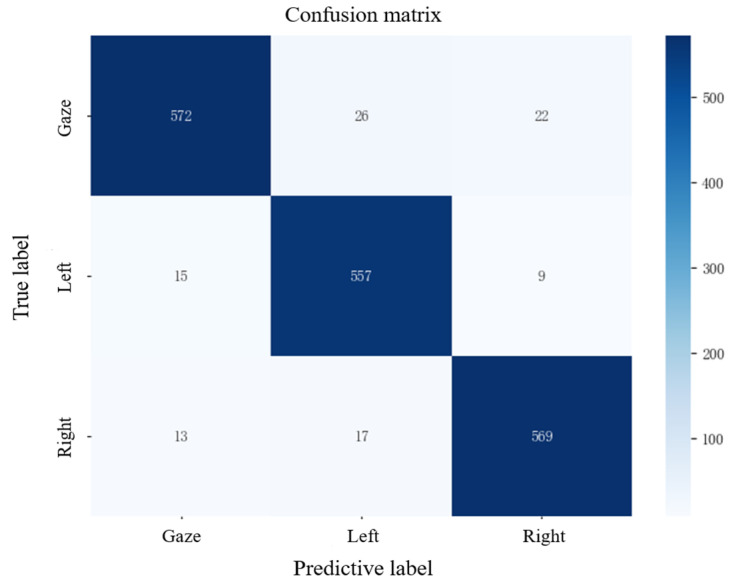
Confusion matrix diagram of discrimination results.

This study employs a combination of frame voting mechanisms and eye movement area discrimination to determine eye movement direction and perform binary value mapping. Experiments were conducted to verify whether the discrimination accuracy of this method meets control requirements. Twenty test subjects were invited to participate in the experiment in the same classroom, with a time window set to 1 s. During the experiment, the test subjects were required to perform 30 trials in the same order of iris movement direction (gaze-left-right), with the eye movement direction discrimination method outputting the results. The test results confusion matrix is shown in Figure 13, with the horizontal axis representing the eye movement direction determined by the method and the vertical axis representing the actual eye movement direction. The accuracy of the method was measured by calculating the ratio of the number of correctly determined eye movement directions to the total number of eye movement directions. The results show that the accuracy for the gaze direction is the highest at 95.3%; the accuracy for the left direction is the lowest at 92.8%; the accuracy for the right direction is 94.8%; and the average accuracy for all three directions is 94.3%.

#### 3.2.2. Eye Movement Coding Matching Experiment

**Figure 14:**
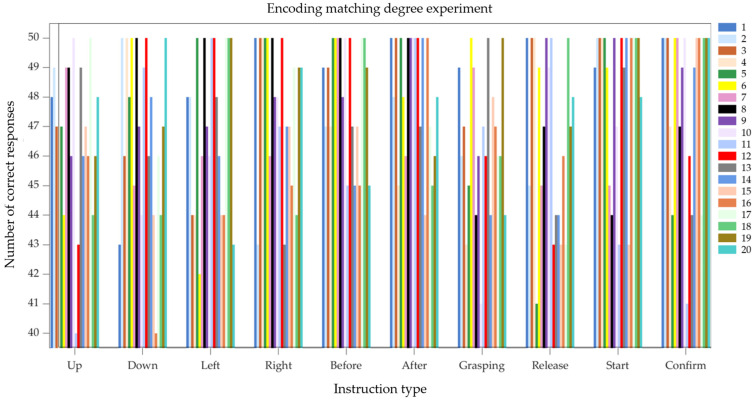
Eye movement coding matching experiment results chart.

To validate the feasibility and matching degree of the eye-tracking encoding used in this study, an eye-tracking encoding matching experiment was conducted. The experimental subjects were the same 20 testers from the previous study. In the experiment, each tester was required to perform the corresponding eye movements for each control command 50 times. The experimental results are shown in Figure 14, where the data from test subjects 1 to 20 are represented by different colors in the figure. The matching degree of the eye-tracking encoding was measured by calculating the ratio of the number of correctly encoded commands to the total number of commands. The average matching degree for all eye-tracking control commands ranged from 93.4% to 96.8%. Among these, the “Start” and “Confirm” commands had the highest average matching degrees at 96.7% and 96.1%, respectively, while the ‘Grab’ and “Release” commands had the lowest average matching degrees at 92.8% and 93.4%, respectively. We analyzed factors potentially affecting test results: the eye-tracking control commands for “grasp” and “release” are “0101” and “1010” respectively. Compared to ‘1111’ and “0011” encoding, these two commands require the iris to move frequently from left to right, making eye-tracking encoding more challenging. Additionally, variations in ambient light can impact the detection model, increasing the error margin in detected coordinate information and consequently reducing the accuracy of eye-tracking encoding.

### 3.3. Eye-Controlled Robotic Arm Experiment

#### 3.3.1. Human–Machine Motion Coordination Experiment

**Figure 15:**
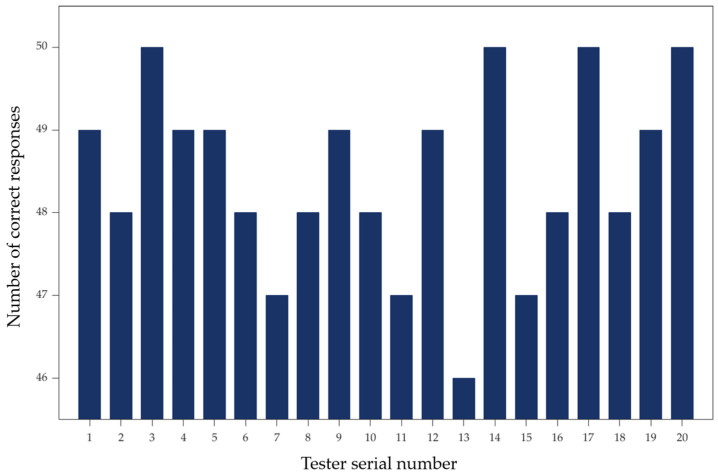
Human–machine collaboration experiment results chart.

To test the overall human–machine coordination during eye-tracking control of a robotic arm and the universality of the control method across different individuals, a human–machine motion coordination experiment was conducted. Each tester performed 50 single-direction movements using eye-tracking control of the robotic arm, with one recorder responsible for documentation. A correct response was recorded when the eye-tracking command matched the actual response of the robotic arm. The human–machine motion coordination was measured by calculating the ratio of correct responses to the total number of attempts. The experimental results are shown in Figure 15, where the horizontal axis represents the tester’s serial number and the vertical axis represents the number of correct responses by the robotic arm. As shown in the figure, the lowest human–machine coordination level calculated for an individual was 92%, the highest was 100%, and the average human–machine coordination level reached 96.5%.

#### 3.3.2. Eye-Controlled Robotic Arm Grasping Experiment

**Figure 16:**
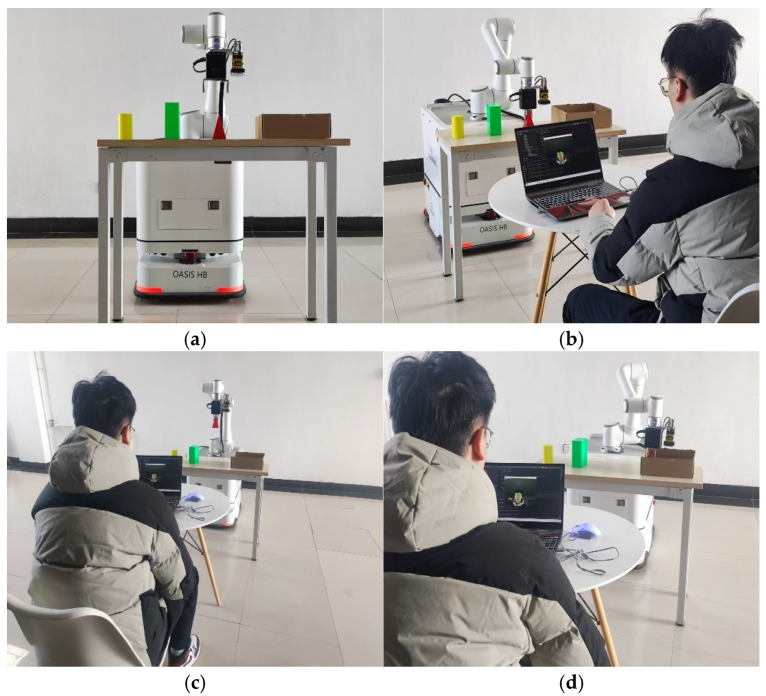
(**a**) Grab the initial position of the experiment; (**b**) Eye tracking control to grab the triangular cone; (**c**) Grab the ascending triangular cone; (**d**) Grab the triangular cone and move it to the target location.

To validate the applicability of the eye-tracking controlled robotic arm platform in real-world environments and its performance in grasping tasks, an eye-tracking controlled robotic arm grasping experiment was conducted. The participants were 20 test subjects who had already mastered the operating methods in previous experiments. Part of the experimental process is shown in Figure 16. Testers used eye-controlled robotic arms to pick up objects and place them into paper boxes. Recorders simultaneously recorded the number of successful picks and the number of eye-controlled commands used for each pick. We will use the ratio of successful picks to total picks to measure the platform’s task completion rate.

Each tester conducted 50 experiments for each of the three grasping tasks: rectangular prism, cylinder, and triangular cone. The experimental results are shown in Table 10. After calculation, in the rectangular prism grasping task, the average completion rate of the testers was 98%, with a maximum of 100% and a minimum of 94%; In the cylindrical object task, the average completion rate was 96%, with a maximum of 100% and a minimum of 90%; in the triangular prism object task, the average completion rate was 78%, with a maximum of 92% and a minimum of 72%. We believe that the lower completion rate for the triangular prism object task may be due to its own shape, resulting in an incompatibility between the robotic gripper and the shape of the object being grasped.

**Figure 17:**
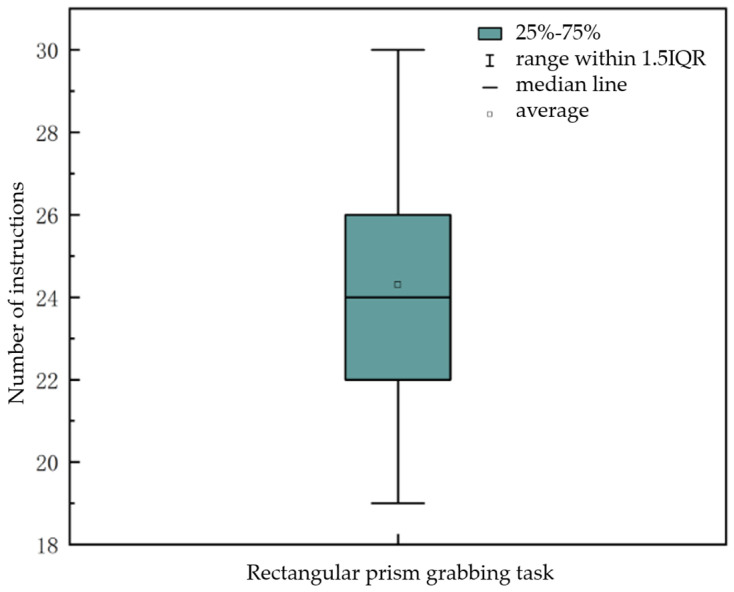
Line box diagram for rectangular block grabbing task.

Since external factors such as the environment, reflections, and object shapes can all influence the eye-tracking control process, to study the applicability of our eye-tracking control method under ideal conditions, we selected the experimental data with the highest completion rate from each tester’s performance in the task of grasping a rectangular block for analysis. As shown in Figure 17, in the rectangular block task, the minimum number of eye-tracking commands used was 19, and the maximum was 30, located at the upper and lower endpoints of the box plot; Based on the upper and lower boundaries of the box plot, where the first quartile (Q1) is 22 and the third quartile (Q3) is 26, it can be seen that 25% of the data is less than or equal to 22, and 75% of the data is less than or equal to 26. This indicates that most rectangular block grasping tasks can be completed within 22 to 26 eye movement commands; The mean value is 24.3, and the median is 24, with a difference of only 0.3 between the two values. This indicates that only a small number of testers used a larger number of commands.

## 4. Conclusions

This study optimized the YOLOv11 model using the EFFM and ORC modules, improving the model’s accuracy in identifying the orbital rim and iris by 1.7% and 9.9%, respectively, and increasing the recall rate by 5.5% and 44%, respectively. Additionally, a method combining frame voting mechanisms with eye movement area discrimination was proposed to address the issue of imprecise eye movement direction identification in traditional methods. After obtaining precise eye movement directions, these were mapped to binary values, and eye movement encoding was used to generate control commands compatible with the robotic arm. Finally, an experimental platform was established to validate the feasibility of the method, achieving average completion rates of 98%, 78%, and 96% for three different grasping tasks.

However, the eye-tracking control method proposed in this study still has limitations. Although the current system performs well in relatively controlled laboratory environments, factors such as significant changes in ambient light and free head movement by test subjects directly impact the quality of input images, becoming the primary sources of error in the control system. Future improvements could incorporate Inertial Measurement Unit (IMU) sensors to compensate for head motion, alongside further optimization of the orbital and iris detection algorithms to adapt to light interference across different time periods. When the number of eye direction images captured within time window N fails to fully characterize the intended eye movement, our eye region area discrimination method may produce misclassifications. Introducing temporal or predictive models to forecast continuous pupil movement could enhance the accuracy of eye direction discrimination. Considering eye-tracking safety, we incorporated a confirmation prompt, which somewhat impacts real-time performance and may cause eye fatigue during testing. Therefore, we can explore hybrid control strategies combining other modalities (e.g., voice, EEG), such as replacing confirmation prompts with error-related potential signals from brain-computer interface technology. This reduces active human involvement, thereby alleviating eye fatigue.

In summary, the eye-tracking control method proposed in this study provides a new solution for improving the quality of life for people with special disabilities and advancing the development of human–computer interaction technology. It demonstrates significant application potential in real-life scenarios. Future work can further optimize the command confirmation process by reducing human active participation to alleviate eye fatigue, thereby solidifying the position of eye-tracking technology in the field of human–computer interaction.

## Figures and Tables

**Table 1:** Eye movement direction discrimination table.

Discrimination Criteria	Eye Movement Direction
$x_{4}<x_{0}\;and\;S_{IR}=0$	left direction
$x_{3}>x_{0}\;and\;S_{IL}=0$	right direction
$x_{3}\leq x_{0}\leq x_{4}\;and\;S_{IL}\geq 1.5S_{IR}$	left direction
$x_{3}\leq x_{0}\leq x_{4}\;and\;S_{IL}\leq 1.5S_{IR}$	right direction
$x_{3}\leq x_{0}\leq x_{4}\;and\;S_{IR}<S_{IL}<1.5S_{IR}$	gaze
$x_{3}\leq x_{0}\leq x_{4}\;and\;S_{IL}<S_{IR}<1.5S_{IL}$	gaze

**Table 2:** Eye movement control command function table.

Serial Number	Robotic Arm Movements	Eye Movement Control Binary Encoding Instructions
1	upward shift	0001
2	downward shift	0010
3	left shift	0100
4	right shift	1000
5	forward shift	0011
6	backward shift	1100
7	grab	0101
8	loosen	1010
9	start	1111
10	confirmation	0000

**Table 3:** Coil position function table.

M528	M529	M530	M531	M532	M533	M534	M535
starting check digit	Write/read data	Get current location	Coil I/O	Coil I/O	Coil I/O	Coil I/O	End check digit

**Table 4:** Coil function code table.

M531~M534	Robotic Arm Movements	Offset Direction Setting
0001	upward shift	Positive direction of the Z-axis
0010	downward shift	negative direction of the Z axis
0100	left shift	negative direction of the X axis
1000	right shift	Positive direction of the X-axis
0011	forward shift	Positive direction of the Y-axis
1100	backward shift	negative direction of the Y axis
0101	grab	- Irrespective of the direction of offset
1010	loosen	Irrespective of the direction of offset

**Table 5:** Experimental environment configuration table.

Experimental Environment	Version Model
operating system	Windows 10 Professional Edition
CPU	12th Gen Intel Core i7-12700F
GPU	NVIDIA GeForce GTX 3060
NVIDIA driver	522.25
CUDA	10.2
compiler	PyCharm2022.1.1
compiled language	Python3.9.19
Deep learning framework	Pytorch2.0.1

**Table 6:** Model comparison experimental data table.

Algorithm	Precision	Recall	mAP@0.5
SSD	0.57	0.513	0.455
Faster R-CNN	0.562	0.528	0.522
YOLO10-n	0.908	0.431	0.489
YOLOv11-n	0.853	0.645	0.719
YOLOv11-n+CA	0.897	0.650	0.745
YOLOv11-n+SE	0.908	0.637	0.760
YOLOv11-n+BiFormer	0.906	0.862	0.907
Our	0.911	0.892	0.920

**Table 7:** Comparative Experiment Table for EFFM Modules.

Algorithm Model	Precision	Recall	mAP@0.5
YOLOv11+CA	0.897	0.650	0.745
YOLOv11+SE	0.908	0.637	0.760
YOLOv11+BiFormer	0.906	0.862	0.907
YOLOv11+EFFM	0.915	0.844	0.910

**Table 8:** Ablation Experiment Data Table.

Algorithm	Category	Precision	Recall	AP (Average Accuracy)
	eye socket	0.901	0.869	0.927
Baseline	iris	0.806	0.421	0.511
	average	0.853	0.645	0.719
	eye socket	0.929	0.93	0.973
Experiment 1	iris	0.901	0.758	0.848
	average	0.915	0.844	0.910
	eye socket	0.925	0.845	0.928
Experiment 2	iris	0.852	0.470	0.591
	average	0.888	0.657	0.759
	eye socket	0.918	0.924	0.970
Experiment 3	iris	0.905	0.861	0.871
	average	0.911	0.892	0.920

**Table 9:** Ablation experiment model parameter table.

Model	Parameters	Gradients	GFLOPs
Baseline	2,590,230	2,590,241	6.4
Experiment 1	2,623,254	2,623,238	6.5
Experiment 2	2,485,174	2,485,158	6.2
Experiment 3	2,516,278	2,516,262	6.3

**Table 10:** Results of the object-grabbing experiment.

Number of Completions	Triangular Cone	Rectangular Prism	Cylinder
maximum	46	50	50
minimum	36	47	45
average	39	49	48

## Data Availability

Data are contained within the article.
